# Making do in the absence of specialist support: Exploring healthcare professionals' views, experiences and behaviours around long‐term post‐bariatric surgery follow‐up care in the United Kingdom

**DOI:** 10.1111/cob.70016

**Published:** 2025-04-29

**Authors:** Ross Watkins, Laura L. Jones, Kenneth Clare, Karen D. Coulman, Colin J. Greaves, Kate Jolly, Emma Shuttlewood, Helen M. Parretti

**Affiliations:** ^1^ Lifespan Health Centre, Norwich Medical School University of East Anglia Norwich UK; ^2^ Department of Applied Health Sciences University of Birmingham Birmingham UK; ^3^ Obesity Institute Leeds Beckett University Leeds UK; ^4^ Obesity UK Halifax UK; ^5^ Bristol Population Health Sciences, Bristol Medical School University of Bristol Bristol UK; ^6^ School of Sport, Exercise and Rehabilitation Sciences University of Birmingham Birmingham UK; ^7^ Specialist Weight Management Service University Hospitals Coventry and Warwickshire NHS Trust Coventry UK

**Keywords:** bariatric surgery, follow‐up, healthcare professionals, interviews, obesity, qualitative

## Abstract

Bariatric surgery is an effective treatment for obesity, but long‐term can lead to health‐related issues. Guidelines highlight the importance of long‐term post‐bariatric surgery follow‐up. However, in the UK, there is currently no specific funding to support the delivery of this care. Our aim was to understand the views and experiences of healthcare professionals (HCPs) around long‐term post‐bariatric surgery follow‐up, and barriers and enablers to care. Semi‐structured interviews with HCPs in UK primary care or specialist weight management services were conducted. The topic guide was theoretically informed by the Capability‐Opportunity‐Motivation‐Behaviour model and the Theoretical Domains Framework. Thematic analysis was undertaken. Twenty‐six HCPs were interviewed. Three core themes were interpreted: *Existing Challenges*, *Mediating Factors* and *Future Directions*. While there was agreement on the need for long‐term support, current provision was variable and hampered by a paucity of referral options. Follow‐up care could be contingent upon the patients' surgical pathway and the culture and expertise within the general practitioner surgery. Participants discussed potential ways to improve care, including using technology, adapting approaches used in other chronic conditions, shared care models and harnessing the potential for peer‐based support to improve wellbeing and quality of life. Healthcare professionals' views and experiences shared in this study highlight the complex issues associated with long‐term bariatric surgery follow‐up. The findings will inform future research to design and implement care pathways that are urgently needed to improve service provision for these patients.

**What is already known about this subject?**

- Current clinical guidance recommends that after bariatric surgery patients should have annual reviews in the long‐term, and in the UK it is suggested that this is delivered using a shared care model between general practitioners (GPs) and specialist weight management services.
- Studies have identified a lack of confidence and competence amongst GPs in delivering long‐term post‐bariatric surgery follow‐up care and current evidence suggests this care is not being delivered.
- Qualitative studies exploring patients' experiences of long‐term follow‐up care after bariatric surgery suggest that patients want more support.

**What this study adds?**

- To the best of our knowledge this is the first qualitative study exploring views and experiences of providing long‐term post‐bariatric surgery care to include both healthcare professionals from a range of professions working within specialist weight management services multidisciplinary teams as well as those working in primary care.
- Our study found that there is frustration and concern amongst these healthcare professionals with the current lack of commissioned services and clear patient care pathways for long‐term post‐bariatric surgery care.
- Our study has identified potential avenues for future intervention development to improve long‐term post‐bariatric surgery follow‐up care and highlights a significant gap in healthcare service provision and a need for further research and policy standards to deliver long‐term post‐bariatric surgery follow‐up care.

## 1 INTRODUCTION

Obesity is a global health priority; in 2022 worldwide 59% of adults were living with overweight or obesity.^1^ In the UK overweight and obesity related ill health is estimated to cost the National Health Service (NHS) £6.1billion/year and is a key target for action in the NHS Long Term Plan.^2^, ^3^ Bariatric surgery is the most clinically‐ and cost‐effective treatment for severe and complex obesity.^4^ Globally, between 507 806 and 598 834 bariatric surgery procedures are performed each year (2020 and 2021 rates), leading to a growing cohort of patients who need care in the longer term.^5^

The health benefits of bariatric surgery are well‐established, including improvements in glucose regulation, cardiovascular disease and overall mortality.^6^ Despite these significant benefits, in the long term, bariatric surgery can result in nutritional deficiencies^7^ and issues such as alcohol/substance misuse can arise.^8^ Although weight loss in the first 2 years post‐surgery is usually significant, from 18 months, weight regain can occur.^9^, ^10^

In the UK, the National Institute for Health and Care Excellence (NICE) recommends specialist follow‐up for 2 years post‐bariatric surgery, then discharge to general practitioners (GPs) for annual reviews under a shared‐care model with a specialist weight management service.^4^ Despite these and other international guidelines highlighting the importance of post‐bariatric surgery follow‐up and the role of primary care, there is no specific funding available to support GPs to undertake annual reviews, and no established services or care pathways to provide long‐term care have been implemented in the UK.^11^, ^12^ There are concerns that many patients are not offered the annual reviews recommended by NICE, and a recent UK study using routinely collected GP data found only 45%–61% of patients had a recorded annual haemoglobin measurement, while blood tests more specific to bariatric surgery were very rare (e.g., copper 0.7%–1.5%).^13^

Several studies have examined the relationships between long‐term follow‐up or nutritional supplement adherence and post‐bariatric surgery complications. A cohort study (*n* = 1160) found that those using specialised bariatric multivitamins at 3 years follow‐up were less likely to develop new deficiencies than non‐users,^14^ while a retrospective study of 431 patients 10 years post‐gastric bypass also found that anaemia was less common in those who had yearly (or in last year) reviews.^15^ Nutritional deficiencies after bariatric surgery can lead to, for example, night‐blindness and cardiomyopathy, and case reports have cited inadequate follow‐up or adherence to supplements as contributing factors for these deficiencies.^16^, ^17^ Lack of follow‐up can also influence weight management post‐surgery, and importantly, weight regain is likely to reduce the health‐related benefits and cost‐effectiveness of bariatric surgery.^18^, ^19^

Supporting patients to engage with post‐bariatric surgery recommendations is vital to optimise outcomes and prevent avoidable harms. Evidence suggests that patients want more support to achieve this and help to manage psycho‐social impacts of bariatric surgery.^20^ Few studies have explored GPs' views and confidence in managing these patients. Most of those conducted have been surveys and suggest that GPs lack confidence and competence in managing patients post‐bariatric surgery.^21^, ^22^, ^23^, ^24^ Effective interventions are needed to support long‐term care for these patients, which are both acceptable to patients, and can be implemented at scale. The aim of this study was to explore in‐depth the views, experiences and behaviours of both primary care and specialist weight management services (SWMS) healthcare professionals' (HCPs) around long‐term post‐bariatric surgery care and to explore barriers and enablers to long‐term post‐bariatric surgery care provision.

## 2 METHODS

Prior to study commencement, a favourable opinion was obtained from the NHS Research Ethics Committee (NorthEast–Newcastle and Tyneside 1REC [23/NE/0039]). NHS management permission was obtained through the Health Research Authority Approval for NHS organisations in England. A patient and public involvement and engagement (PPIE) advisory group (PAG) was established and involved throughout the study.

### 2.1 Study design and setting

Semi‐structured one‐to‐one interviews with UK primary care and SWMS HCPs were conducted using web conferencing software, Microsoft Teams.

### 2.2 Recruitment, sampling and consent

GP participants were recruited via three Clinical Research Networks. Both GPs and SWMS HCPs (e.g., bariatric surgeons, physicians, psychologists, dietitians, nurses and pharmacists) were recruited via professional organisations (e.g., British Obesity and Metabolic Surgery Society [BOMSS], Royal College of General Practitioners and Association for the Study of Obesity) via their social media/membership emails and newsletters/websites, professional networks, social media and snowball sampling.

An expression of interest form was used to obtain contact details and information about roles and experience, which facilitated purposive sampling based on clinical profession, years of experience, geographical region, age, ethnicity and role within public (NHS) and/or private sector. Those sampled were contacted by the research team directly to discuss the study further, provided with a participant information leaflet and given an opportunity to ask questions. If willing to proceed, the researcher (RW) arranged for them to be sent a consent form to complete electronically. Once consent was obtained, an interview was scheduled. Each participant was assigned a unique study reference number.

### 2.3 Data collection

One researcher (RW) undertook all interviews using Microsoft Teams between July and August 2023. The interviews lasted on average 52 min (range 45–60 min). The interviews focused on participants' views and experiences of delivering post‐bariatric surgery follow‐up care, using a topic guide (see Supporting Information S1) developed by the research team (academics with experience in obesity research, qualitative methodology and health psychology as well as clinicians working in primary care and SWMS), and our PAG. A combination of the Capability‐Opportunity‐Motivation‐Behaviour (COM‐B) model and the Theoretical Domains Framework (TDF) was used to inform the topic guide to allow us to ensure our investigation was systematic and comprehensive in informing the data collection.^25^, ^26^ COM‐B allowed us to explore key influences on important behaviours and system‐level influences while the TDF allowed us to identify in more detail influences on HCP behaviours related to implementation of post‐bariatric surgery care. The topic guide prompts underwent minor modifications in light of early interview data, consistent with an inductive approach.^27^ Prompts broadly focussed on HCPs' experiences and views on managing patients' long‐term post‐bariatric surgery, their knowledge and skills in managing these patients and thoughts on how future care could be improved. Interviews were audio‐recorded and transcribed verbatim.

### 2.4 Data analysis

Codebook thematic analysis^28^ was used to analyse transcripts, supported by NVivo software (QSR International; Version 12 Pro) to help organise, code and explore the data. The analytic focus was to organise the data in a meaningful way according to the a priori aims of the study, and to allow identification of topics and issues of importance to participants. Repeated reading and familiarisation with the data led to the development of an initial set of codes. These codes were assimilated into a coding hierarchy. As new and recurring themes were identified, the hierarchy was refined and used to inform the development of an overarching conceptual model. The themes and their names and explanations were continually refined through discussion between the researchers RW, HMP and LLJ to ensure that they were distinct from other themes, internally coherent and consistently applied. Initial interpreted themes and findings were discussed with our PAG and KC. The study is reported to reflect the Standards for Reporting Qualitative Research (SRQR) criteria^29^ (see Supporting Information S1).

### 2.5 Reflexivity statement

RW is a researcher with over 5 years postdoctoral experience in qualitative health services research (White British male), LLJ is an experienced mixed methods applied health researcher (White British female) and HMP is an academic GP with expertise in obesity (White British female). The wider study team comprised a mix of genders, ages and professional expertise (e.g., clinical psychologist, dietitian, health psychologist, expert by experience).

## 3 RESULTS

**FIGURE 1:**
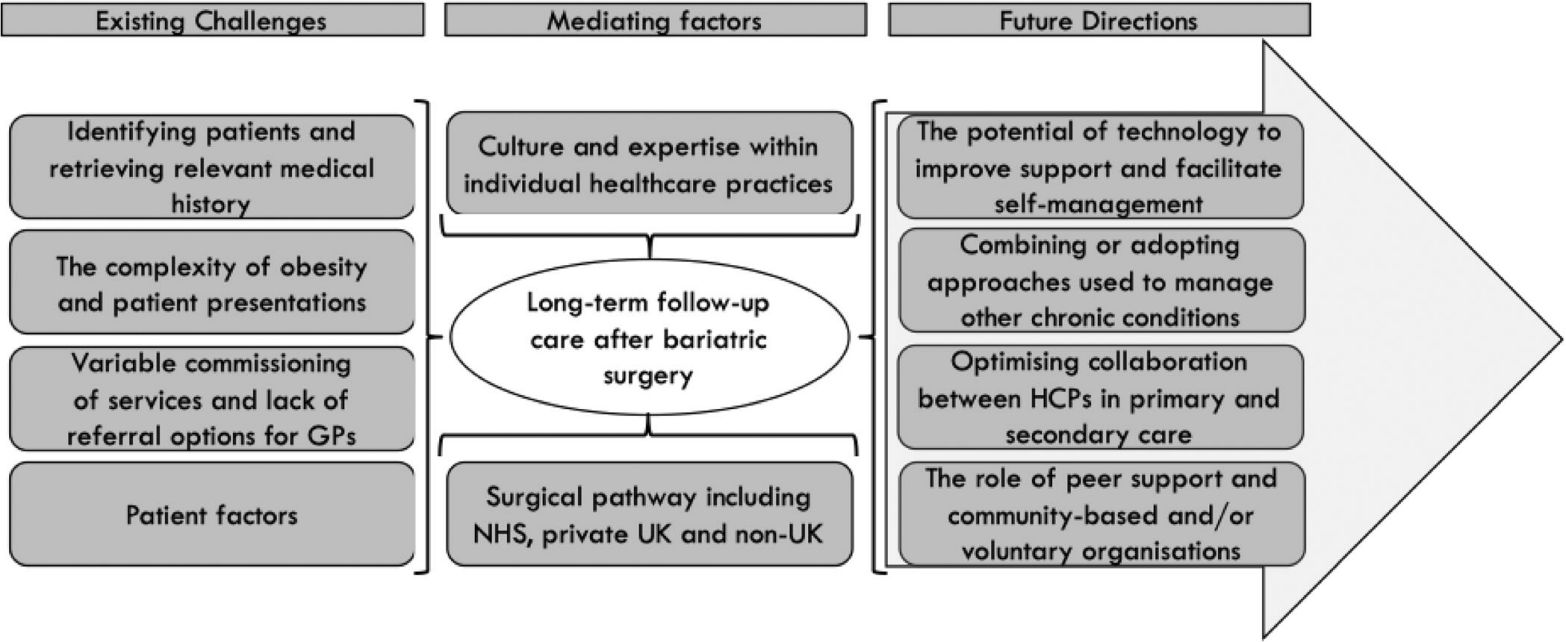
Conceptual model of the themes related to providing long‐term post‐bariatric follow‐up. GPs, general practitioners; HCPs, healthcare professionals; NHS, National Health Service.

Interviews were completed with 26 HCPs (Table 1). Ten overlapping and inter‐related themes related to providing long‐term post‐bariatric follow‐up were grouped into three distinct core themes: (1) *Existing Challenges*, (2) *Mediating Factors* and (3) *Future Directions*. Figure 1 summarises these in a conceptual model.

**TABLE 1:** Healthcare professional participant characteristics.

Characteristic	*n* = 26
Clinical profession	
GP	10
Bariatric Dietitian	5
Bariatric Clinical Psychologist	4
Bariatric Pharmacist	2
Bariatric Surgeon	2
Bariatric Physician	2
Bariatric Nurse	1
Years in role	
1–5 years	5
6–10 years	6
11–15 years	7
16–20 years	5
21–25 years	1
26 years +	2
Provider	
NHS	22
Private/combined	4
Geographical region	
Southeast	6
Southwest	6
Northeast	6
West Midlands	3
East of England	2
Scotland	1
Wales	1
Age	
30–39 years	6
40–49 years	13
50–59 years	5
60–69 years	2
Ethnicity	
White	22
Black Caribbean	1
Black African	1
Indian	1
Chinese	1

Abbreviations: GP, general practitioner; NHS, National Health Service.

### 3.1 Existing Challenges

#### 3.1.1 Identifying patients and retrieving relevant medical history

For NHS patients, participants described how treatment information was routinely coded into GP electronic patient records following surgery, patients were typically formally discharged into primary care after 2 years follow‐up under the bariatric surgery team with a formal discharge letter detailing the surgical procedure and follow‐up information: *When they're discharged at two years, they're discharged back to the GP with a letter, which details our whole plan of care, and then after that what's expected from the GPs* (HCP18, Nurse).

However, for patients who had surgery privately, post‐operative follow‐up was variable, with minimal follow‐up reported in most cases, and sometimes limited to post‐operative in‐patient recovery only. Information from private hospitals about the surgical procedure and recommended follow‐up was shared with GPs on an ad hoc basis, and often divulged by the patient rather than the surgical team, a situation which was described as *really disturbing* by one GP participant (HCP23, GP). Information gaps were particularly prominent when surgery was undertaken privately abroad: *The private ones are a real, that's a nightmare because often they [the patient] have very poor information and sometimes, they have it in a foreign language, they don't know what operation they've had* (HCP17, GP).

#### 3.1.2 The complexity of obesity and patient presentations

HCPs described the challenges of managing physical and mental health conditions that patients can present with following bariatric surgery, including *hypoglycaemia, food aversions or food intolerances, difficulties eating, eating disorder symptoms, … and alcohol addiction* (HCP21, Psychologist), as well as issues related to surgery, for example, excess skin or where surgery was deemed to be unsuccessful. Participants discussed how patients may have a legacy of unresolved psychological issues that could re‐emerge, such as disordered eating, or that for some patients new disorders emerge: *I would say a re‐emergence, or an emergence, of disordered eating, so sometimes you see sort of binge eating and compulsive eating returning, but then I would also say that sub‐group of people who go on to develop sort of anorexic type eating or bulimia* (HCP13, Psychologist). Participants described the need for multidisciplinary input: *There are going to be times when you think “I have no idea what's happening” and I'm not afraid to ask for support from surgical colleagues, medical colleagues, dietitians, psychologists, whoever I need to help support it because it is a major complex disease and it takes everybody to have a think about what happens* (HCP03, Physician).

#### 3.1.3 Variable commissioning of services and lack of referral options for GPs

There was a consensus that patients should have access to follow‐up in primary care. However, participants reported that the potential to deliver this support was variable among commissioning areas in terms of provision and availability of referral pathways.

*There are patients who need a lot more support and it, even in the NHS […], they just haven't got anybody to refer them to* (HCP04, Dietitian).

There was a paucity of referral options more broadly, with participants citing a lack of specialist dietetic and psychological support referrals available to primary care. This resulted in GPs having to make do in the absence of specialist support, often referring to non‐specialist community‐based support. However, there were concerns this might not meet the needs of patients: *it just feels like so much of the presentation gets missed or isn't understood in those [community based] services* (HCP13, Psychologist). Further to this, there was concern that referrals would not be accepted by community‐based services because they fell outside of their scope: *I have primary care dietitians, but I'm not sure that they would accept a post‐bariatric patient, they would be, ‘no, we can't accept that in primary care, that must go to the dietetics, the post‐bariatric clinic at the hospital’* (HCP11, GP). Concerns about the lack of clear referral pathways were also raised by some SWMS HCPs: *And they go through the cracks because nobody will see them* (HCP07, Dietitian).

Participants also reported that treatment was typically only available for more extreme presentations: *Let's say somebody develops an eating disorder, they will only be seen by [the] NHS if they are extremely, extremely acute, otherwise they are just left with eating disorder charities and the online chat. Because there is nothing else* (HCP07, Dietitian).

#### 3.1.4 Patient factors

The inequity of accessing follow‐up care was widely discussed by participants who described how internalised stigma prevented some patients from seeking support in primary care, particularly if they had surgery abroad and had not previously been supported through an NHS bariatric surgical pathway.

*Some of the people we know are going to struggle are the ones who experience the most internalised weight stigma and shame, which then prevents them seeking help if they need it. They're going to be the ones who won't reach out because they're so embarrassed, they'll feel like they've let the service down, they've failed the surgery, this was their last chance* (HCP21, Psychologist).

In some cases, participants suggested that patients can be reluctant to reveal that they had undergone surgery abroad, fearing judgement by HCPs as well as friends and family. It was proposed that how patients perceived their surgery could impact on their understanding of long‐term follow‐up care *they perhaps don't see it as medical, they see it as cosmetic and not really related […] and I think sometimes see it in that respect and don't understand the implications and requirements for monitoring and if the surgeon who's done the surgery hasn't made that clear to them* (HCP14, GP). Interviewees also described the role of socioeconomic status in shaping access to post‐bariatric support: *I've got patients who are on the poverty line, or below the poverty line who won't be able to afford to drive over to [county name], it's just not going to happen, so I could refer them but there's no way they'd be able to go to appointments, especially not repeated, frequent appointments* (HCP02, GP). The geographical range in which some services operated made in‐person appointments impractical, particularly if patients did not drive or had difficulties leaving the house: *Wait an hour between ambulance drop off and their appointment, and wait another hour after the appointment for an ambulance to take them home, it's six to eight hours kind of lost, so for these patients* (HCP07, Dietitian).

### 3.2 Mediating Factors

#### 3.2.1 Culture and expertise within individual healthcare practices

There was an acknowledgement that some HCPs may not prioritise support for people living with obesity or those seeking support after bariatric surgery and that this had the potential to affect the culture within individual practices around follow‐up care.

*There's still people who have very mixed feelings about people who struggle with their weight or who don't agree with bariatric surgery and it's hard, you know, that we know the HCPs also have these biases and I think that does play a role in why it hasn't been given enough priority* (HCP17, GP).

Interviewees posited that this could be due to a reticence to consider obesity as a chronic disease, and that this fundamentally affected treatment: *they think it's just a lifestyle symptom, as opposed to a disease* (HCP10, Nurse) and *I think there's a general attitude amongst GPs that these are people who are doing things they shouldn't do to their health, not taking responsibility … So that's all underlying it* (HCP14, GP). In contrast, prioritising weight management and post‐bariatric support was regarded a crucial gateway to the management of risk factors for a host of other chronic diseases: *There are so many areas of primary care, diabetes, cardiovascular disease, stroke, cancer, that are so impacted by this, and I spend so much of my life chatting about HRT [Hormone Replacement Therapy] to women, and talking about HRT increases your risk of breast cancer by three or four in a thousand, but actually obesity will double your risk of breast cancer, I just don't think we treat it very well at all*. (HCP11, GP).

This theme also touched on the awareness of post‐bariatric surgery support amongst HCPs and the knowledge and experience (or lack) of HCPs in delivering support. For example, assessing micronutrient status required specialist knowledge and this could affect a GP's propensity to investigate: *The feedback I've had from our community GPs is that they feel they're lacking the knowledge, and they want more specialist input, and that's why they don't want to do bloods or they're more reluctant to do them* (HCP10, Nurse). The skills required to provide long‐term support to patients were thought to have a crucial bearing on patients' experiences of receiving care. This provision of care was contingent on training and guidance needed to upskill and inform HCPs in primary care and acceptance of the need to draw on specialist resource and/or expertise in secondary care: *It needs to be part of the GP vocational training scheme, very specifically, not just sort of scattered throughout, there needs to be a specific curriculum section on obesity, and a separate one on lifestyle medicine* (HCP14, GP).

#### 3.2.2 Surgical pathway including NHS, private UK and non‐UK

Patients' experience of receiving long‐term support was often based on whether their surgery had been undertaken on the NHS or privately. The lack of funding and access for routine follow‐up care in the NHS, either in primary care or specialist settings, for those who had private surgery was described.

*We basically had to say if you've had surgery privately abroad, we will no longer accept a referral for regular routine [NHS] follow up, so we'll only accept you into the service if you are having problems* (HCP06, Dietitian).

*The GPs certainly in my regional area are telling me that they're not funded to follow any of these [private] patients up, … so there is a huge issue about having their yearly annual blood checks that they should have done* (HCP10, Nurse).

Participants discussed how HCPs applied discretion around follow‐up care, with some choosing to refer for tests and/or treatment despite there being no funding for these services: *I think we have a duty of care, if we've received a letter, and it's in NICE guidance, we've received a letter this person needs follow up we have to do it, we've got no option. Yeah, it'd be nice if it was funded but it's our job unfortunately, you know, we can't get paid for everything* (HCP20, GP). Others cautioned against the implementation of long‐term routine follow‐up, suggesting that given the limited resources available, follow‐up support should be applied judiciously: *This has massive implications for cost, so if people, if GPs, were to fully implement the BOMSS nutritional guidelines tomorrow, I dare say all GP practices will go bankrupt in this country* (HCP19, Surgeon).

### 3.3 Future Directions

#### 3.3.1 The potential of technology to improve support and facilitate self‐management

Despite concerns over NHS Information Technology infrastructure and ability to integrate new systems, interviewees recognised the potential for technology to enhance communication between HCPs and patients, enable more efficient use of resources, and provide greater support to patients in self‐monitoring and self‐managing their health. Participants described how this could include sending annual review reminders, sharing blood test results, providing patients with symptom‐relevant information, and supporting behaviour change: *I think apps and a lot of online resources can be really amazing for just actually getting a snapshot of where you are, where is your mood, what are you actually eating …* (HCP09, Psychologist).

In addition, it was suggested that app‐based or virtual support could reduce the time burden on HCPs in primary care, which was cited as a major barrier to the provision of support. Some interviewees cautioned that the use of technology had the potential to marginalise those who may not be able or willing to use it. However, others thought that it had the potential to increase engagement and enhance support for some patients who might find it difficult to access traditional modes of support: *Sometimes the people who've experienced a lot of trauma, they find trust and communication and talking and opening up really hard. So, giving them a way to do that via message or just a different way where they don't have a person staring at them, … I actually think it'd be a helpful thing. We have quite a few people with neurodiversity issues, but again there are ways of adapting technology to meet their needs* (HCP21, Psychologist).

#### 3.3.2 Combining or adopting approaches used to manage other chronic conditions

Participants discussed the potential for follow‐up care to mirror the support given for other chronic conditions, such as diabetes, where patients are automatically recalled for an annual review to help ensure that their condition remains well managed: *If it was diabetes, or cardiac disease, or depression, then we would have systems for reminding them, but because its bariatric surgery, it's not given that priority, so we don't have systems to remind them* (HCP02, GP).

Although advised in NICE guidance, this type of annual review is not currently commissioned for post‐bariatric surgery follow‐up, and interviewees argued that due to a lack of funding and possible stigma, this type of review would be unlikely to be conducted routinely: *We're not commissioned to do it, we have to get through the work that we are commissioned to do, and we are paid to do, so who is going to do all these things?* (HCP02, GP). Participants also discussed the possibility of combining post‐bariatric surgery follow‐up with annual reviews for any concomitant co‐morbidities, potentially reducing both patient and HCP burden. However, concerns were raised that clinicians would find this challenging: *It's quite hard as a clinician to have more than one hat on when you're thinking about that person's problem. So I think, that probably, if you tried to combine the two, you'd end up with somebody maybe doing a good job of the diabetes but forgetting about the bariatric side of things, or doing a good job of bariatric and having to rush through the diabetes* (HCP02, GP).

#### 3.3.3 Optimising collaboration between HCPs in primary and secondary care

This theme encompassed the notion of *shared care* (HCP15, GP) where follow‐up support is jointly managed between primary care and SWMS, along with the involvement of the patient. In practice, this could mean that when patients are discharged into primary care 2 years' post‐surgery they could be referred back into specialist services if required (e.g., to see a specialist dietitian or psychologist). Alternatively, one participant advocated for the secondment of specialists in the community: *I think we need to get the hospital specialists out of hospitals and into community, so follow up should be in the community, but by the same team that looked after them originally, seeing them less often, in a different context … because I think that it'd be good for the specialist teams to see how people live for the rest of their life with this, as opposed to just seeing them for like two years* (HCP15, GP). Implicit within this would be the ability to provide a flexible package of support according to an individual's specific needs rather than taking a one‐size‐fits‐all approach: *It's something about being able to be flexible to the person's needs, … because everybody is experiencing it differently, actually the specifics of where they are what they're struggling with, that's the bit that needs a bit of space to be tailored to the individual* (HCP22, Psychologist). For a shared care model to be practicable, participants emphasised the need for a written shared care agreement to be in place.

#### 3.3.4 The role of peer support and community‐based and/or voluntary organisations

Participants widely acknowledged the important role of peer and community‐based support in enhancing the wellbeing and quality of life of patients, particularly in the long‐term, when the *honeymoon period* following surgery begins to wane and some patients may begin to experience weight regain and the associated impact on mental health: *creating a space in which people can work through some of the impulse control and self‐esteem type issues that, eventually, are long‐term and on‐going, after that kind of initial buzz has gone from the rapid weight loss* (HCP09, Psychologist).

Peer support was perceived as a means of alleviating the sense of a *cliff‐edge* (HCP04, Dietitian) when patients are discharged from specialist care. Shared lived experience, and the opportunity to share experiences with others who had been through what they had was seen as life affirming: *You can maximise your chances of having a good life when there are others around you that kind of get it* (HCP09, Psychologist). However, participants suggested that these support groups should be underpinned by experts in the field or be moderated by HCPs to ensure the veracity of content and/or that any concerning disclosures within the group were appropriately managed: *I think Obesity UK have a moderated Facebook group with people with lived experience of surgery who also have professionals, so there are some forums, so that's where I would direct people to if they wanted some peer support in the longer‐term* (HCP22, Psychologist).

*I think patient support, peer support, however you phrase it, that has a lot more power than me talking at patients: patients listen a lot more to other patients, the only care you need to exercise there, is that, you know, sometimes good information gets disseminated, which is good, but sometimes bad information gets disseminated in peer support groups so we need some training for those people who are going to be supporting patients* (HCP19, Surgeon).

## 4 DISCUSSION

Interviews with a diverse group of HCPs working both in primary care and across the SWMS multidisciplinary team (MDT) identified 10 themes related to experiences and opinions on the provision of long‐term follow‐up support after bariatric surgery. There was agreement among HCPs in this study that patients should receive long‐term follow‐up in primary care after bariatric surgery, but the importance of having appropriate funding in place to support this was also raised by participants. It was also felt that providing this long‐term support for patients' post‐bariatric surgery was contingent upon capacity to manage follow‐up in primary care as well as access to specialist support. In the absence of funded specialist referral options, there were concerns about both the appropriateness and feasibility of referring to community services. It was widely acknowledged that a shared care approach was needed, and it was proposed that peer support could contribute to improving patient wellbeing, particularly in the current absence of funded healthcare support.

Our findings resonate with a 2019 review of patients' experiences of long‐term care post‐bariatric surgery, as well as a subsequent qualitative study, which highlighted the need for better care beyond 2 years post‐surgery. In these studies, patients described a ‘*honeymoon period*’ for the first 2 years post‐surgery, followed by feeling ‘*abandoned*’ when discharged.^20^, ^30^ Patients also expressed a desire for review by clinicians with specialist expertise. This was echoed in this study, with HCPs also expressing a need for specialist input and support.

Relatively few studies have explored GPs' views and confidence in managing post‐bariatric surgery patients. A survey study conducted in Scotland reported that 76% of GPs were not comfortable managing patients post‐bariatric surgery.^21^ A similar picture was seen from survey studies conducted in the US, Canada and France.^22^, ^23^, ^24^ A qualitative study published in 2023 aimed to understand what is needed to support long‐term management of patients post‐bariatric surgery in community‐based settings. Participants included HCPs (including five GPs) and patients from a single region in the UK.^31^ Some of our findings are aligned with those identified in this study such as a frustration with a lack of framework for delivering long‐term care and lack of primary care HCPs' knowledge to deliver the care. A qualitative study by Jose et al. set in Tasmania also explored the role of GPs in the surgical management of obesity. Although this study only focused on the management of patients who had laparoscopic adjustable gastric bands (a procedure now infrequently performed in the UK) it included findings relevant to this study: a lack of clarity of the GP role post‐surgery and a need for greater collaboration between GPs, surgeons and patients.^32^ Together, these studies suggest that GPs lack confidence and competency to manage patients post‐bariatric surgery and are aligned with our findings. To our knowledge, only one previous qualitative study has explored views and experiences of SWMS HCPs on long‐term post‐bariatric surgery. This study interviewed 15 bariatric surgeons in Germany and highlighted the heterogeneity of healthcare delivery in bariatric surgery in Germany post‐operatively.^33^

### 4.1 Strengths and limitations

This study has included a diverse sample of HCPs with experience of managing patients after bariatric surgery across the UK. To the best of our knowledge, this is the first qualitative study exploring long‐term post‐bariatric surgery care to include both HCPs working in primary care and from a range of professions within the SWMS MDT (both private and NHS providers). It is key to understand these different perspectives given that collaboration between these HCPs will likely be key to developing an effective future service. Since there is significant geographical variability in the commissioning of services across the UK, it was also important that we included HCPs from a wide range of geographical areas and their experiences. The topic guide for this study was theoretically informed by COM‐B and the TDF as well as including input from the range of expertise within the research team and our PAG.^25^, ^26^ As a team, when we looked at the data, we chose not to analyse against COM‐B and TDF domains as they were too rigid given the breadth and complexity of the behaviours identified. We chose to analyse inductively to ensure all elements were appropriately interpreted. A limitation of this study was that all of the participants in this study worked and treated patients in the UK healthcare system. However, there is potential for our findings to be transferable to other healthcare systems, indicated by the alignment of findings with those from the studies by Breuing and Jose (set in Germany and Australia, respectively).^32^, ^33^

### 4.2 Conclusions

Despite working in different settings and geographical areas across the UK, our research found similarities in experiences and views amongst our participants, with HCPs expressing frustration at the current lack of commissioned services, clear patient care pathways, and in some circumstances, concerns around patient safety. Our findings highlight a significant gap in healthcare service provision and an urgent need for national policy standards on the commissioning and delivery of long‐term post‐bariatric surgery care.

An important aspect of this study was exploring participants' views and ideas on how care could be improved in the future, and this study has identified several potential targets for future intervention(s) to deliver more effective care for these patients. The chronic nature of obesity and the need for lifelong follow‐up after bariatric surgery pose a difficult challenge for resourcing support in terms of HCP time, the cost of tests and any referral for further treatment. For example, the sustained long‐term behavioural change needed post‐bariatric surgery is a significant challenge, particularly given that individuals may continue to be exposed to factors that contributed to the development of their obesity. However, it is also important that consideration is given to the health cost of not providing patients with sufficient long‐term follow‐up support. Given limited resources, alternative approaches to the management of follow‐up care may need to be considered. For example, annual reviews could be combined with those commissioned to manage other chronic conditions, such as diabetes or cardiovascular disease. Technology could be employed to encourage self‐monitoring and facilitate self‐management. The potential for peer or community groups to provide long‐term support may also offer promise. These now need to be explored in further research.

## AUTHOR CONTRIBUTIONS

**HMP**: conceptualisation, data curation, formal analysis, funding acquisition, project administration, validation, visualisation, writing—review and editing. **RW**: data curation, formal analysis, investigation, validation, visualisation, writing—original draft, writing—review and editing. **ES**, **KDC**, **KC**, **KJ** and **CJG**: conceptualisation, funding acquisition, writing—review and editing. **LLJ**: conceptualisation, formal analysis, funding acquisition, validation, visualisation, writing—review and editing. All authors were involved in writing the paper and had final approval of the submitted and published versions.

## CONFLICT OF INTEREST STATEMENT

Helen M Parretti and Emma Shuttlewood are BOMSS council members and have organised educational events supported by Ethicon for BOMSS members (honoraria received for educational events). Helen M Parretti has developed an algorithm for the management of obesity in primary care (with accompanying supplement, video and conference presentation) which was supported by arm's length sponsorship from Novo Nordisk (honoraria received). Helen M Parretti was a member of the steering group for the Obesity Empowerment Network and the NICE obesity clinical guidelines (NG246) committee and is a member of the NICE quality standards committee. Karen D Coulman is the BOMSS research co‐lead for dietetics and a member of the scientific advisory board for Oxford Medical Products. Kenneth Clare is a director of bariatric surgery, Obesity UK, and chair of the European Coalition for People Living with Obesity, a trustee for the Association for the Study of Obesity, and has been a member of a Novo Nordisk Disease Experience Expert Panel since 2017 and a Patient Advisory Board member for Boehringer Ingelheim since 2020 (honoraria received for these last two roles).

## Supporting information


**Data S1.** Supporting Information.
