# Supplementary material for: Making do in the absence of specialist support: Exploring healthcare professionals' views, experiences and behaviours around long‐term post‐bariatric surgery follow‐up care in the United Kingdom
Source: Clin Obes. 2025 Apr 29;15(4):e70016. doi: 10.1111/cob.70016 (PMC12289401; doi:10.1111/cob.70016)
Supplement: Supplementary file 1 — Data S1. Supporting Information. [file COB-15-e70016-s001.pdf]

Improving the long-term care of patients who have had bariatric surgery: PROMISE CARE study

Work package 1 Healthcare Professional Interviews
Discussion Guide
Chief Investigator: Dr Helen Parretti

Please note: *this interview discussion guide will be a flexible tool, open to revision if new areas of interest arise during the process of data collection and as the study progresses. Further, in order to adapt to any logistical factors (e.g. limited time for an interview) it is not essential that each interview should include every line of questioning as detailed below. Depth of exploration of fruitful areas of discussion is more important than complete coverage of all areas in every interview.*

Below are **example questions only,** they are not necessarily in the order they will be asked; the final discussion guides will be informed by literature search, research team discussion and input from our PPIE contributors prior to the interviews.

Introduction

- Welcome/introductions
- Thank the participant(s) for agreeing to take part
- Review the purpose of the study in general
- Emphasise the value of their views and opinions – there are no right or wrong answers
- Ensure that the consent form has been read and understood
- Statement on confidentiality, right to withdraw consent, recording of the discussion
- Ask if the participant(s) have any questions before starting the interview/discussion
- Switch on audio recording (either dictaphone or using recording functionality in the online platform (e.g. Microsoft Teams/Zoom)) and start interview, remind that main focus is long-term care post-bariatric surgery (>2 years post-surgery, the time usually discharged from NIHS bariatric surgery services)

Objective		Potentially related TDF domains (to use as prompts if needed)
To explore experiences of managing patients long-term post bariatric surgery	Capability (physical & psychological)	
	Explore their experience(s) of managing patients long-term post-bariatric surgery:<br>- what have been your experiences of managing these patients with acute problems/complications?<br>- what have been your experiences of managing the routine care and monitoring of these patients with?<br>- can you give examples of positive/negative experiences?	Social/professional role and identity<br>Knowledge<br>Skills<br>Environmental context and resources<br>Emotion

	Opportunity (physical & social)	
	- how do you identify patients as having had bariatric surgery?<br>- how often do you see patients who have had bariatric surgery?<br>- has the number of patients you are seeing post-surgery changed over time? (and why do you think it has changed?)<br>- what have been the presenting problems/needs of these patients? (e.g. acute complication, routine follow-up monitoring)	Knowledge<br>Skills<br>Environmental context and resources
	Motivation (reflective & automatic)	
	Reflecting on your experiences, how does this make you feel about managing bariatric surgery patients long-term in the future?	Emotion
To explore HCPs knowledge and skills around managing patients long-term post-bariatric surgery.	Capability (physical & psychological)	
	Explore their knowledge and confidence about facilitators/barriers to the management and care of patients long-term post-bariatric surgery<br>- How confident do you feel about your knowledge and skills in managing patients long-term post bariatric surgery?<br>- How confident are you in your knowledge and skills of managing routine monitoring in long-term post bariatric surgery care?<br>- How confident are you in your knowledge and skills in managing acute problems or complications post bariatric surgery?<br>- Do you feel that you have adequate knowledge of the latest guidance documents relevant for supporting patients post bariatric surgery in the long-term?	Beliefs about capabilities<br>Knowledge<br>Skills
	Opportunity (physical & social)	
	- What has been your experience of accessing any additional guidance	Knowledge<br>Skills

	to support your knowledge and skills in this area?<br>- What has been your experience of accessing any specific training in this area?<br>- What have been the main barriers to developing your knowledge and skills in managing patients long-term post bariatric surgery?<br>- What has supported you or helped you develop knowledge and skills in this area?	Environmental context and resources<br>Emotion
	Motivation (reflective & automatic)	
	- What do you think routine care should look like for these patients?<br>- What do you think acute care/care of complications should look like for these patients?	Beliefs about consequences<br>Environmental context and resources
	Check and clarify	
	Do you have any questions about what we have talked about so far?<br><br>Is there anything else you think is important that you would like to add at this point?	
To explore barriers and facilitators to delivering long-term post-bariatric surgery care.	Capability (physical & psychological)	
	Explore views on support and training to enable delivery of long-term post-bariatric surgery care:<br>- Are there any skills/education/training that you think would help you to manage patients post-bariatric surgery?<br>- What else could support you to manage patients post-bariatric surgery?<br>(With prompts for acute or routine care)	Skills<br>Knowledge<br>Reinforcement<br>Environmental context and resources
	Opportunity (physical & social)	
	Explore any factors that might make it difficult to deliver post-bariatric surgery care<br>- What makes it difficult for you to provide effective long-term care to post bariatric surgery patient? e.g. work environment,	Environmental context and resources<br>Skills<br>Knowledge<br>Beliefs about capabilities<br>Beliefs about consequences

	colleagues, expertise, specialist support, capacity, geographical distance to bariatric unit, confidence, knowledge, personal thoughts and feelings<br><br>Explore any factors that might make it easier to deliver post-bariatric surgery care<br>- Can you think of anything that would help you/make it easier for you to care for these patients? e.g. work environment, colleagues, geographically close bariatric unit, support from specialists, commissioning, training, personal motivation?<br>(With prompts for acute or routine care)	Social/professional role and identity<br>Emotion
	Motivation (reflective & automatic)	
	- How would/do you feel about delivering routine post-bariatric surgery care to patients?<br>- Is there any difference between how you feel about delivering this care to patients who have had privately surgery compared with those who have had NHS funded surgery?<br>- How would/do you feel about managing complications post-bariatric surgery including acute problems?	Beliefs about capabilities<br>Beliefs about consequences<br>Social/professional role and identity<br>Environmental context and resources<br>Emotion<br>Intentions
	Check and clarify	
	Do you have any questions about what we have talked about so far?<br><br>Is there anything else you think is important that you would like to add at this point?	
To explore views on what long-term post-bariatric surgery care should/could look like.		
	- What do you think long-term post-bariatric surgery care should include and why? e.g. content of annual reviews (including psychology), self-management,	Beliefs about capabilities<br>Beliefs about consequences<br>Social/professional role and identity<br>Environmental context and resources

	peer support, specialist input, acute complications<br>- In which setting(s) do you think long-term post-bariatric surgery care could or should be delivered, and why?<br>(With prompts for acute or routine care)<br>- Which healthcare professionals would be most appropriate to be delivering long-term care of patients post-bariatric surgery, and why?<br>(With prompts for acute or routine care)<br>- Who should hold clinical responsibility for long-term care after bariatric surgery, and why?	Intentions<br>Knowledge<br>Skills
	- When/if the post bariatric surgery care has been delivered well, what do you think has supported or enabled this?<br>- Have you experienced any successful examples of the delivery of long-term follow-up care after bariatric surgery?	Environmental context and resources
	- What are your thoughts on self-management for bariatric patients in the longer term?<br>- How else could patients be supported following bariatric surgery?	
	Check and clarify	
	Do you have any questions about what we have talked about?<br><br>Is there anything else you think is important that you would like to add?	

Closing

- End of the interview

- Check/clarify points back with the interviewee as necessary
- Explain where they could find the results of the study
- Reminder about confidentiality, right to withdraw and snowballing for participants
- Remind participant reimbursement for their time is available on request
- Thank them for their time
- File a copy of the completed consent form and send a copy to the participant

Standards for Reporting Qualitative Research (SRQR)*

http://www.equator-network.org/reporting-guidelines/srqr/

Page/line no(s).

Title and abstract

**Title** - Concise description of the nature and topic of the study Identifying the study as qualitative or indicating the approach (e.g., ethnography, grounded theory) or data collection methods (e.g., interview, focus group) is recommended	1
**Abstract** - Summary of key elements of the study using the abstract format of the intended publication; typically includes background, purpose, methods, results, and conclusions	4

Introduction

**Problem formulation** - Description and significance of the problem/phenomenon studied; review of relevant theory and empirical work; problem statement	5-6
**Purpose or research questio**n - Purpose of the study and specific objectives or questions	6

Methods

**Qualitative approach and research paradigm** - Qualitative approach (e.g., ethnography, grounded theory, case study, phenomenology, narrative research) and guiding theory if appropriate; identifying the research paradigm (e.g., postpositivist, constructivist/ interpretivist) is also recommended; rationale**	6-8
**Researcher characteristics and reflexivity** - Researchers' characteristics that may influence the research, including personal attributes, qualifications/experience, relationship with participants, assumptions, and/or presuppositions; potential or actual interaction between researchers' characteristics and the research questions, approach, methods, results, and/or transferability	9
**Context** - Setting/site and salient contextual factors; rationale**	6
**Sampling strategy** - How and why research participants, documents, or events were selected; criteria for deciding when no further sampling was necessary (e.g., sampling saturation); rationale**	6
**Ethical issues pertaining to human subjects** - Documentation of approval by an appropriate ethics review board and participant consent, or explanation for lack thereof; other confidentiality and data security issues	6
**Data collection methods** - Types of data collected; details of data collection procedures including (as appropriate) start and stop dates of data collection and analysis, iterative process, triangulation of sources/methods, and modification of procedures in response to evolving study findings; rationale**	7-8

**Data collection instruments and technologies** - Description of instruments (e.g., interview guides, questionnaires) and devices (e.g., audio recorders) used for data collection; if/how the instrument(s) changed over the course of the study	8 and Supplementary Material
**Units of study** - Number and relevant characteristics of participants, documents, or events included in the study; level of participation (could be reported in results)	9 and Table 1
**Data processing** - Methods for processing data prior to and during analysis, including transcription, data entry, data management and security, verification of data integrity, data coding, and anonymization/de-identification of excerpts	7-9
**Data analysis** - Process by which inferences, themes, etc., were identified and developed, including the researchers involved in data analysis; usually references a specific paradigm or approach; rationale**	8-9
**Techniques to enhance trustworthiness** - Techniques to enhance trustworthiness and credibility of data analysis (e.g., member checking, audit trail, triangulation); rationale**	8-9

Results/findings

**Synthesis and interpretation** - Main findings (e.g., interpretations, inferences, and themes); might include development of a theory or model, or integration with prior research or theory	9-18
**Links to empirical data** - Evidence (e.g., quotes, field notes, text excerpts, photographs) to substantiate analytic findings	9-18

Discussion

**Integration with prior work, implications, transferability, and contribution(s) to the field -** Short summary of main findings; explanation of how findings and conclusions connect to, support, elaborate on, or challenge conclusions of earlier scholarship; discussion of scope of application/generalizability; identification of unique contribution(s) to scholarship in a discipline or field	18-21
**Limitations** - Trustworthiness and limitations of findings	19-20

Other

**Conflicts of interest** - Potential sources of influence or perceived influence on study conduct and conclusions; how these were managed	21
**Funding** - Sources of funding and other support; role of funders in data collection, interpretation, and reporting	22

*The authors created the SRQR by searching the literature to identify guidelines, reporting standards, and critical appraisal criteria for qualitative research; reviewing the reference lists of retrieved sources; and contacting experts to gain feedback. The SRQR aims to improve the transparency of all aspects of qualitative research by providing clear standards for reporting qualitative research.

**The rationale should briefly discuss the justification for choosing that theory, approach, method, or technique rather than other options available, the assumptions and limitations implicit in those choices, and how those choices influence study conclusions and transferability. As appropriate, the rationale for several items might be discussed together.

Reference:
O'Brien BC, Harris IB, Beckman TJ, Reed DA, Cook DA. **Standards for reporting qualitative research: a synthesis of recommendations.** *Academic Medicine*, Vol. 89, No. 9 / Sept 2014
DOI: 10.1097/ACM.0000000000000388
